# Plasma-derived phosphoglycerate mutase 5 as a biomarker for Parkinson’s disease

**DOI:** 10.3389/fnagi.2022.1022274

**Published:** 2022-10-28

**Authors:** Liang Feng, Haijun He, Xi Xiong, Kai Xia, Shuangjie Qian, Qianqian Ye, Feifei Feng, Shuoting Zhou, Xianchai Hong, Yiming Liu, Chenglong Xie

**Affiliations:** ^1^Department of Neurology, Qilu Hospital of Shandong University, Jinan, China; ^2^Department of Neurology, The First Affiliated Hospital of Wenzhou Medical University, Wenzhou, China; ^3^Key Laboratory of Alzheimer's Disease of Zhejiang Province, Zhejiang, China; ^4^Institute of Aging, Wenzhou Medical University, Wenzhou, Zhejiang, China; ^5^Oujiang Laboratory, Wenzhou, Zhejiang, China

**Keywords:** PGAM5, Parkinson’s disease, mitophagy, receiver operating characteristic, AUC

## Abstract

**Background:**

We aimed to examine whether plasma-derived phosphoglycerate mutase 5 (PGAM5) can be a biomarker for Parkinson’s disease (PD) diagnosis as well as its association with the severity of motor/non-motor manifestations of PD.

**Methods:**

We enrolled 124 patients with PD (PD group) and 50 healthy controls (HC group). We measured plasma PGAM5 levels using a quantitative sandwich enzyme immunoassay. Patients with PD underwent baseline evaluations using the Unified Parkinson’s Disease Rating Scale (UPDRS), while participants in both groups were evaluated using scales for non-motor manifestations. Receiver operating characteristic curves were used to evaluate the predictive utility of plasma PAMG5 alone and combined with other factors.

**Results:**

Plasma PAMG5 levels were significantly higher in the PD group; the area under the curve (AUC) of plasma PGAM5 levels alone was 0.76. The AUC values for elderly participants and patients without hypertension were 0.78 and that for was 0.79. Notably, plasma PGAM5 levels combined with plasma oligomeric α-synuclein (α-syn) and the score of the REM sleep behavior disorder questionnaire-Hong Kong (RBDQ-HK) showed AUC values of 0.80 and 0.82. Multivariable logistic analysis revealed that plasma PAMG5 levels were independently associated with PD (odds ratio,1.875 [95% confidence interval 1.206–2.916], *p* = 0.005) but not the severity of motor/non-motor manifestations of PD.

**Conclusion:**

Plasma PGAM5 is an independent biomarker for PD, especially among elderly patients (age > 60 years) and patients without hypertension. The predictive utility of PGAM5 was improved when combined with plasma oligomeric α-syn or the RBDQ-HK score.

## Introduction

Parkinson’s disease (PD) is among the most common neurodegenerative disorders, which affects approximately 1% of individuals aged >60 years (de Lau and Breteler, 2006). Bradykinesia, rest tremor, and rigidity are the core PD symptoms; moreover, it is important to consider non-motor PD manifestations, including emotional disorders, autonomic dysfunction, and REM sleep behavior disorder (Postuma et al., 2015; Schapira et al., 2017). The characteristic pathological changes in PD include the loss of dopaminergic neurons in the substantia nigra pars compacta (SNpc) as well as the presence of Lewy bodies (LB) containing aggregates of α-synuclein (α-syn) and other neuronal compounds (Wakabayashi et al., 2013). There is increasing evidence suggesting that genetic and environmental factors, especially mitochondrial dysfunction, may be crucially involved in PD pathophysiology (Giannoccaro et al., 2017; Ammal Kaidery and Thomas, 2018). Maintenance of mitochondrial homeostasis and cellular energy supply relies on normal mitochondrial function, including a healthy mitochondrial network, natural mitochondrial metabolism, and repair of mitochondrial DNA damage (Lo and Hannink, 2006; Chaikuad et al., 2017; Cheng et al., 2021). Dopaminergic neurons are metabolically active, which demand high mitochondrial energy. Moreover, insufficient clearance of damaged mitochondria, especially mitophagy dysfunction, is an important pathophysiological characteristic of PD (Hou et al., 2020). The aggregation of α-syn could result from impaired autophagic-lysosomal degradation (Moors et al., 2016; Hou et al., 2020).

This study aimed to examine the relationship between phosphoglycerate mutase 5 (PGAM5) and PD. PGAM5 is a mitochondrial membrane protein with specific Ser/Thr/His protein phosphatase activity and is involved in regulating mitochondrial dynamics, typically in mitophagy (Yu et al., 2020; Baba et al., 2021; Cheng et al., 2021), while mitophagy plays an active role in maintaining cell function (Palikaras et al., 2018). Cellular stress models have shown that PGAM5 is involved in the link of the PTEN-induced putative protein kinase 1 (PINK1) and Parkin with the ubiquitin pathway as well as a receptor-mediated signaling loop with FUNDC1 (Chen et al., 2014; Lu et al., 2014; Wu et al., 2014). PGAM5 is critical for mitochondrial fission through dynamin-related protein 1 dephosphorylation. PGAM5 deletion increases reactive oxygen species (ROS) levels (Park et al., 2018; Yu et al., 2020). Even moderate ROS breaks the KEAP1 (Kelch ECH associating protein 1)-PGAM5 complex, which leads to KEAP1 degradation and PGAM5 accumulation, mitophagy would be promoted (Zeb et al., 2021, 2022). A previous study disclosed that PGAM5-deficient mice presented with a Parkinson’s-like movement phenotype, and PGAM5 exerted cytoprotective effects on dopaminergic neurons (Lu et al., 2014). Based on the above research, in this study, we aimed to examine whether plasma PGAM5 could be a diagnostic biomarker for PD or a predictive biomarker for the motor and non-motor manifestations of PD.

## Materials and methods

### Study design and participants

All participants were recruited from the First Affiliated Hospital of Wenzhou Medical University. Patients with PD were classified as having clinically established or probable PD based on the Movement Disorder Society Clinical Diagnostic Criteria (Postuma et al., 2015). Healthy controls (HC) comprised clinical patients or inpatients without a history of Parkinson’s syndrome, psychiatric disorders, brain trauma or stroke, and cancer.

Participants were analyzed using the Unified Parkinson’s Disease Rating Scale (UPDRS) (Fahn and Elton, 1987) and Hoehn–Yahr staging (Hoehn and Yahr, 1967) to evaluate the motor symptoms of PD. The Chinese version of the Mini-Mental State Examination (MMSE) was used for cognitive examination, with adjustment of the cutoff score for cognitive impairment according to the years of education as follows: illiterate, ≤17 points; primary school education, ≤20 points; and postsecondary education or above, ≤24 points (Folstein et al., 1975; Katzman et al., 1988; Yao et al., 2010; Cui et al., 2011). Examination of emotional aspects was performed using the Hamilton Depression Rating Scale (HAMD) and Hamilton Anxiety Rating Scale (HAMA). Specifically, depression and anxiety were defined as HAMD score ≥17 points and HAMA score ≥14 points, respectively. The REM sleep behavior disorder questionnaire-Hong Kong (RBDQ-HK) was used to detect REM sleep behavior disorder (RBD), with a cutoff value of ≥18 points (Li et al., 2010).

### Blood collection and testing of PAMG5

Plasma samples were obtained through blood centrifugation (3,000 ×*g* for 10 min) and stored at −80°C until subsequent analysis. Plasma PGAM5 and oligomeric α-syn levels were determined using enzyme-linked immunosorbent assay (Jianglai Biotechnology Company, Shanghai, China; No: JL11208, JL41188, respectively) following the manufacturer’s instructions. The samples were processed by a laboratory technician blinded to all clinical data. Subsequently, 80 μl of standard solution and 20 μl of samples (5 × diluted) were pipetted into the wells of 96-well plates. Next, 100 μl of antibody-horse radish peroxidase conjugate (anti-PGAM5 antibody and oligomeric α-syn antibody; MyBioSource, United States) was added to standard and sample wells, which were covered using an adhesive strip and incubated for 60 min at 37°C. After washing four times, the plates were incubated in tetramethylbenzidine substrate for 15 min at 37°C; additionally, the reactions were stopped using 2 mol/l H_2_SO_4_. The plates were read at the 450-nm wavelength. All samples were run in duplicate.

### Statistical analysis

Continuous variables were assessed for normality using the Kolmogorov–Smirnov test. Normally distributed variables were compared using Student’s *t*-test and were expressed as the mean ± standard deviation (SD). Non-normally distributed variables were compared using the Mann–Whitney *U*-test and were expressed as the median (interquartile range, IQR). Categorical variables were compared using the Chi-square test or Fisher’s exact test and were presented as numbers (percentages). Two-tailed Spearman’s rank tests were used for correlation coefficients. Non-normal dependent variables underwent Box-Cox transformation using Python software (Python 3.8) with linear regression. A receiver operating characteristic (ROC) curve was used to evaluate the predictive utility of plasma PAMG5 alone or combined with other factors. Logistic regression analysis was used for multivariable analysis, with the results being expressed as the adjusted odds ratios (OR) with the corresponding 95% confidence intervals (CIs). Statistical analyses were performed using GraphPad Prism software (version 8) and SPSS (version 25.0). Statistical significance was set at a two-tailed *p* < 0.05.

## Results

**Figure 1:**
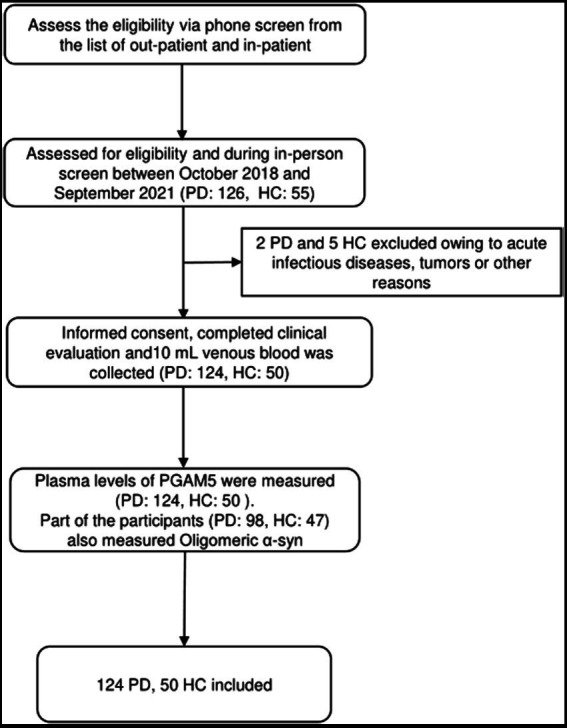
The screening process of participants.

We assessed 126 patients with PD and 55 HC for eligibility during in-person screening from October 2018 to September 2021. Among them, we included 174 participants (124 patients with PD and 50 HC) after excluding two patients with PD and five HC due to having acute infectious diseases, comorbid tumors, or other reasons. Figure 1 shows the screening process for the participants. There were no significant between-group differences in age, sex, body mass index, years of education, and MMSE scores. The HAMD (*p* < 0.01), HAMA (*p* < 0.01), and RBDQ-HK (*p* < 0.01) scores were higher in the PD group than in the HC group. Table 1 summarizes the demographic characteristics and scores of non-motor manifestations of all participants, as well as the UPDRS I-IV scores and Hoehn–Yahr stages in the PD group. The median plasma PAMG5 level was significantly higher in the PD group than in the HC group (864.79 pg/ml [IQR: 794.07–961.85] vs. 780.32 pg/ml [IQR: 686.57–825.16]). Since previous studies have shown that oligomeric α-syn is a potential diagnostic biomarker for PD (El-Agnaf et al., 2006; Tokuda et al., 2010), we examined plasma levels of oligomeric a-syn in most participants (PD group, *n* = 98; HC group, *n* = 47). The median oligomeric a-syn levels were significantly higher in the PD group than in the HC group (2.86 ng/ml [IQR: 2.54–3.47] vs. 2.42 ng/ml [IQR 2.25–2.78]).

**Table 1:** Basic demographics and clinical characteristics of the participants.

Characteristics	Control (*n* = 50)	PD (*n* = 124)	*p*-value
Male, *n* (%)	21 (42.00)	63 (50.80)	0.293
Age, median (IQR), years	66.00 (60.00, 69.25)	65.00 (60.00, 71.00)	0.968
BMI, median (IQR), kg/m^2^	24.14 (22.79, 25.83)	23.90 (21.97, 26.01)	0.781
Education, median (IQR), years	4.00 (0, 5.25)	5.00 (0, 7.00)	0.189
Duration, median (IQR), years	-	4 (2.00, 7.00)	-
H&Y stages, median (IQR)	-	2 (1.50, 3.00)	-
UPDRS, median (IQR)	-	38.50 (27.00, 53.00)	-
UPDRS-I, median (IQR)	-	2.00 (1.00, 4.00)	-
UPDRS-II, median (IQR)	-	11.00 (8.00, 16.00)	-
UPDRS-III, median (IQR)	-	23.00 (13.00, 33.00)	-
UPDRS-IV, median (IQR)	-	2.00 (0, 4.00)	-
MMSE, median (IQR)	23.00 (20.00, 26.25)	24.00 (18.00, 27.00)	0.898
HAMD, median (IQR)	3.00 (0, 5.00)	6.00 (3.00, 10.00)	<0.001^*^
HAMA, median (IQR)	3.50 (1.75, 9.00)	10 (5.25, 14.75)	<0.001^*^
RBDQ-HK, median (IQR)	4.00 (2.75, 8.00)	19.5 (5.00, 42.25)	<0.001^*^
ADL, median (IQR)	20.00 (20.00, 20.00)	26.00 (21.00, 33.00)	<0.0001^*^
PGAM5, median (IQR), pg/ml	780.32 (686.57,825.16)	864.79 (794.07, 961.85)	<0.001^*^
Oligomeric α-syn (ng/ml^a^)	2.42 (2.25, 2.78)	2.86 (2.54, 3.47)	<0.001^*^

Data are expressed as mean, median, or *n* (%). IQR, interquartile range; PD, Parkinson disease; UPDRS, unified Parkinson’s disease rating scale; MMSE, Mini-Mental State Examination; HAMD, Hamilton Depression Scale; HAMA, Hamilton Anxiety Scale; RBDQ-HK, REM sleep behavior disorder questionnaire-Hong Kong; ADL, Activity of Daily Living Scale; BMI, Body Mass Index; PGAM5, phosphoglycerate mutase 5.

a 47HC and 98PD also examined plasma oligomeric α-syn.Asterisk (*) indicates statistical significance (*P* < 0.05).

**Figure 2:**
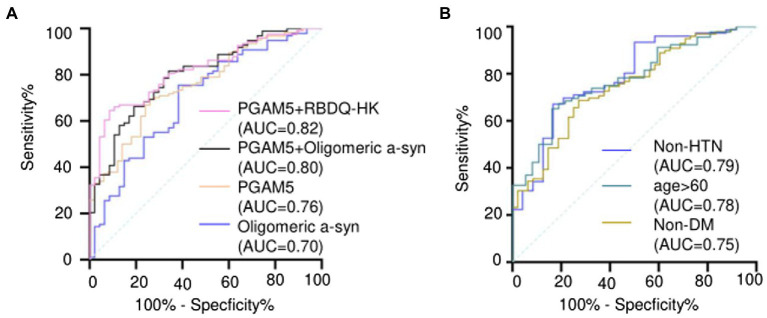
**(A)** ROC curves and AUC of plasma PAMG5 combined with the score of RBDQ-HK or plasma oligomeric α-syn; and ROC curves and AUC of plasma PAMG5 or plasma oligomeric α-syn separately; **(B)** ROC curves and AUC of plasma PAMG5 in different PD subgroups.

ROC curve analysis revealed that the area under the curve (AUC) values for plasma PGAM5 and oligomeric α-syn were 0.76 (95% *CI*, 0.68–0.83) and 0.70 (95% *CI*, 0.68–0.83), respectively. Notably, the combination of plasma PAMG5 levels with plasma oligomeric α-syn levels and the RBDQ-HK scores improved the diagnostic utility (AUC values: 0.80 [95% *CI*, 0.72–0.87] and 0.82 [95% *CI*, 0.75–0.88], respectively; Figure 2A). To determine whether plasma PAMG5 levels differed according to the PD subgroups, the participants were divided into hypertension (HTN) and non-HTN subgroups (hypertension was defined if patients had a history of hypertension or were using antihypertensive medication) and diabetes mellitus (DM) and non-DM subgroups (diabetes mellitus was defined by a history of DM or were using oral hypoglycemic agents/insulin). There were 76 PD patients and 24 HC in non-HTN subgroup, while there were 48 PD patients and 26 HC in HTN subgroup, and there were 99 PD patients and 48 HC in non-DM subgroup, while there were 25 PD patients and 2 HC in DM subgroup; subjects were also divided into different subgroups according to the age (age ≤50, 50 < age ≤ 60, 60 < age ≤ 70, age >70). The results are shown in Table 2. The AUC values for elderly participants (>60 years old) and patients without hypertension were 0.78 (95% *CI*, 0.69–0.86, Figure 2B) and 0.79 (95% *CI*, 0.68–0.89, Figure 2B), respectively.

**Table 2:** PGAM5 levels in subgroups of hypertension (HTN), diabetes mellitus (DM), and age.

PGAM5 (pg/ml)	PD median (IQR)	Control mean (IQR)	*p*-value
Non-HTN (nPD = 76, nHC = 24)	860.48 (790.38, 942.36)	750.97 (665.49, 812.99)	<0.0001^*^
HTN (nPD = 48, nHC = 26)	875.11 (795.53, 1044.50)	788.09 (733.43, 857.71)	0.0008*
Non-DM (nPD = 99, nHC = 48)	860.55 (793.59, 945.94)	780.32 (679.37, 831.07)	<0.0001^*^
DM (nPD = 25, nHC = 2)	899.66 (797.93, 1061.87)	757.17 (707.95, 806.38)	0.114
Age ≤50 (nPD = 7, nHC = 1)	932.52 (858.47, 1045.67)	690.16	NR
50 <age ≤ 60 (nPD = 25, nHC = 12)	839.76 (792.42, 983.55)	823.02 (679.41, 892.18)	0.1166
60 < age ≤ 70 (nPD = 56, nHC = 26)	871.77 (789.38, 961.85)	760.87 (672.88, 807.61)	<0.0001^*^
Age >70 (nPD = 36, nHC = 11)	849.23 (785.25, 953.77)	786.80 (756.30, 813.55)	0.0407^*^

Data are expressed as mean, median, or *n* (%). IQR, interquartile range; HTN, hypertension; DM, diabetes mellitus; non-HTN, non-hypertension; non-DM, non-diabetes mellitus.Asterisk (*) indicates statistical significance (*P* < 0.05).

To determine whether plasma PAMG5 levels were independently associated with PD, we initially performed a univariate analysis followed by a multivariable analysis using logistic regression analysis (Table 3). Univariate analysis revealed significant between-group differences in the presence of HTN, DM, anxiety, and RBD. Moreover, the plasma levels of PAMG5 and oligomeric α-syn (both graded in quartiles) showed significant between-group differences. However, multivariable analysis revealed that PD showed an independent association with DM (*OR*, 23.67; 95% *CI*, 2.26–246.33; *p* = 0.008), anxiety (*OR*, 10.61; 95% *CI*, 2.05–54.82; *p* = 0.005), RBD (*OR*, 3.43; 95% *CI*, 1.07–11.04; *p* = 0.039), plasma PAMG5 levels (*OR*, 1.88; 95% *CI*, 1.21–2.92; *p* = 0.005), and plasma oligomeric α-syn (*OR*, 1.67; 95% *CI*, 1.07–2.60; *p* = 0.023).

**Table 3:** Univariable and multivariate analysis on plasma PGAM5 and oligomeric α-sync levels and other clinical characteristics in logistic regression.

Variables	Univariable analysis				Multivariable analysis			
	*β*	Wald *χ*^2^	*p*-value	*OR* (95% *CI*)	*β*	Wald *χ*^2^	*p*-value	*OR* (95% *CI*)
Age	−0.05	0.09	0.76	0.95 (0.70–1.30)				
Sex	0.44	1.45	0.23	1.55 (0.76–3.14)				
BMI	0.03	0.54	0.46	1.03 (0.95–1.13)				
HTN	−0.85	5.46	0.02	0.43 (0.21–0.87)	−0.93	3.60	0.058	0.40 (0.15–1.03)
DM	2.34	5.01	0.03	10.35 (1.34–80.08)	3.16	7.01	0.008	23.67 (2.28–246.33)
Cognitive impairment	0.72	2.58	0.11	2.05 (0.85–4.92)				
Depression	0.91	0.67	0.42	2.47 (0.28–21.79)				
Anxiety	2.34	9.64	0.002	10.41 (2.37–45.69)	2.36	7.94	0.005	10.61 (2.05–54.82)
RBD	2.21	18.45	0.0001	9.12 (3.33–24.99)	1.23	4.27	0.039	3.43 (1.07–11.04)
PGAM5	0.85	19.93	0.0001	2.34 (1.61–3.40)	0.63	7.79	0.005	1.88 (1.21–2.92)
Oligomeric α-syn	0.59	11.41	0.001	1.798 (1.28–2.53)	0.51	5.15	0.023	1.67 (1.07–2.60)

The cutoff point of the MMSE for cognitive impairment was adjusted according to years of education. Patients who were illiterate according to the level ≤17 points, and a primary school education according to the level ≤20 points, postsecondary education or above according to the level ≤24 points; Hamilton Depression Rating Scale (HAMD) and the Hamilton Anxiety Rating Scale (HAMA) for emotion examination; depression was defined when HAMD score ≥17 points; and anxiety was defined as a HAMA score ≥14 points. The RBDQ-HK rating scale was used to detect whether subjects with a REM sleep behavior disorder (RBD) were defined when the RBDQ-HK score was ≥18 points. BMI, body mass index; plasma levels of PAMG5 and oligomeric α-syn were both graded in quartiles (grade 1–4).

**Figure 3:**
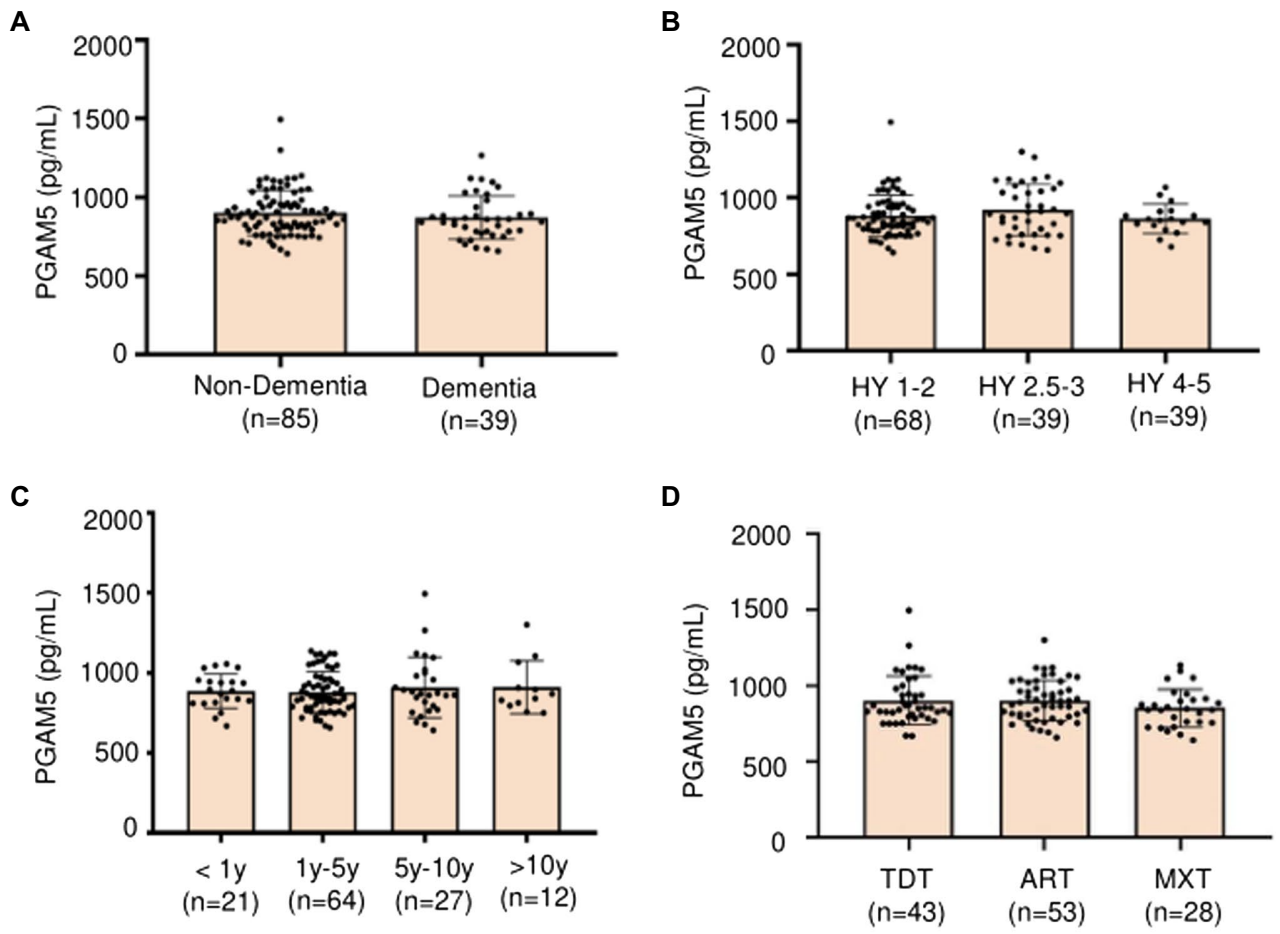
**(A)** Plasma PAMG5 in different statuses of cognitive function (Non-dementia and dementia); **(B)** Plasma PAMG5 of different degrees of Hoehn–Yahr stage; **(C)** Plasma PAMG5 in different duration of PD; **(D)** Plasma PAMG5 in different subtypes of PD (divided as tremor-dominant type, akinetic-rigid type, and mixed type).

**Figure 4:**
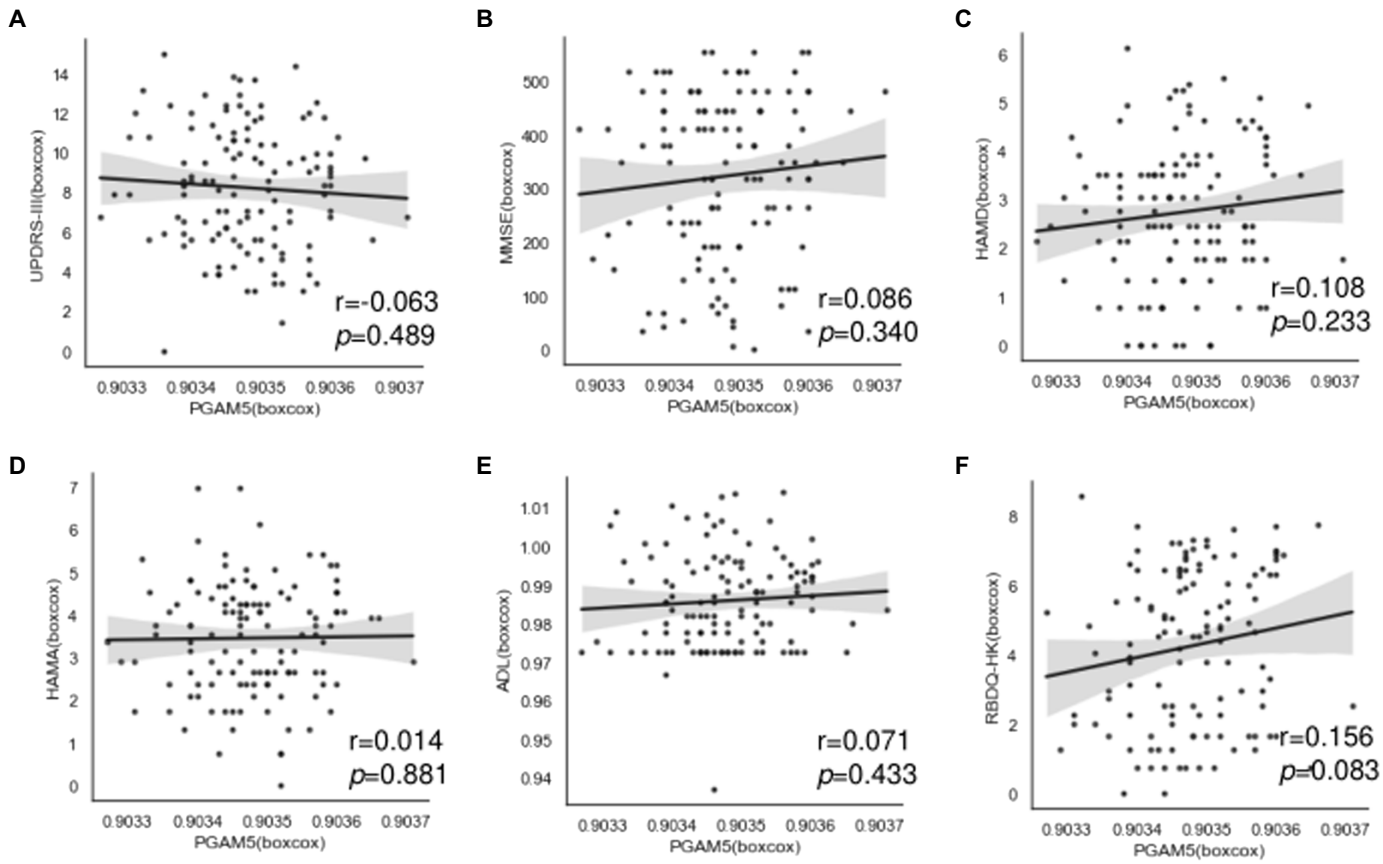
Correlation between plasma PAMG5 levels and scores of motor symptoms or non-motor manifestations in PD group, including: **(A)** UPDRS part III; **(B)** MMSE; **(C)** HAMD; **(D)** HAMA; **(E)** RBDQ-HK; **(F)** ADL (*r* and *p* got by Pearson test, after Box-Cox transformed).

We performed further analysis regarding the correlation of plasma PAMG5 levels with the severity of motor or non-motor manifestations of PD. We observed no significant correlation of plasma PAMG5 levels with the cognitive function status, disease severity (Hoehn–Yahr staging), disease duration (≤1 year, 1–5 years, 5–10 years, and >10 years), and PD subtypes (tremor-dominant type, akinetic-rigid type, mixed type; Iijima et al., 2011; Figure 3). Spearman’s correlation analysis revealed a correlation between plasma PGAM5 levels and RBDQ-HK scores (*r* = 0.183, *p* = 0.044). However, linear regression analysis revealed no correlation of the plasma PGAM5 levels with the motor PD symptoms (UPDRS-III score) or non-motor PD manifestations (Figure 4).

## Discussion

To our knowledge, our study is the first study discussing the correlation between plasma PGAM5 levels and clinical features of PD. PGAM5 is a mitochondrial membrane protein; recent studies suggest that PGAM5 mainly resides in the inner mitochondrial membrane, and is critical for mitochondrial homeostasis by regulating mitophagy (Chen et al., 2014; Wu et al., 2014; Park et al., 2018). Mitophagy occurs not only in a pathological state but also in the normal state. PGAM5 would be cleaved and released from mitochondria, then translocate to the cytosol (Baba et al., 2021); PGAM5 was most easily cleaved by the PARL (presenilin-associated rhomboid-like protein) compared with other substrates, especially in cases of cellular stress (Sekine et al., 2012; Lysyk et al., 2021). The exact mechanism by which PGAM5 is released to blood is not clear. In the present research, plasma PGAM5 levels were detected both in PD group and HC group, and we found that plasma PAMG5 levels were significantly higher in PD group compared with HC. The reasons why plasma PGAM5 levels were ascending in PD patients are worth exploring. On the one hand, there may be a negative feedback mechanism of PGAM5 levels during a certain PD course, especially on the early stage, for increased PGAM5 levels may improve mitochondrial function and reduce mitochondrial damage due to neuronal degeneration (Cheng et al., 2021). The study on PGAM5-knockout mice showed Parkinson’s-like movement disorders involving dopaminergic neurodegeneration in the SNpc and the abnormal behavioral phenotype was rescued by L-DOPA (Lu et al., 2014). On the other hand, ascending PGAM5 level may be a signal of aging. A current study on the connection of PGAM5 and reproductive cell showed that PGAM5 was highly expressed and positively associated with aging; a total of 70 patients were recruited in this clinical study. As the researcher described, when PGAM5 is eliminated, the mitochondrial function and metabolism of aging cells are partially reversed (Li et al., 2022). In our study, plasma PGAM5 levels were higher among elderly participants (>60 years) in PD group compared with HC group, this result also indicates that PGAM5 may have some association with aging. Few articles discussed the association of PGAM5 with chronic clinical diseases, most studies were based on cell models or experimental animal models. A diabetic model of mice showed that mitochondrial fission and the expression of PGAM5 were increased, which aggravated the development of diabetic renal tubular injury (Liu et al., 2020). One research used autoimmune encephalomyelitis (EAE) models announced silence of PGAM5 in spinal cord ameliorated the disease severity in the mice, and overexpression of PGAM5 reactivated cellular necroptosis and inflammation in microglia (Wang et al., 2018). In our study, plasma PGAM5 levels were different in subgroups of Non-HTN, HTN, and Non-DM, but not in DM subgroup; as for the limitation of the sample size, these conclusions need further confirmation.

In summary, the mechanism whether elevated PGAM5 levels act as a protective factor by mitophagy or will do harm to neuron through mitochondrial fission and necrosis remains controversial. Nevertheless, our findings confirm the relationship between PGAM5 and PD, and point out that plasma PGAM5 levels may be a valuable diagnostic biomarker for PD. As rapid eye movement (REM) sleep behavior disorder (RBD) is not only an important symptom of prodromal PD but also a typical non-motor manifestations of PD (Berg et al., 2015; Schapira et al., 2017; Seppi et al., 2019), the diagnostic utility of plasma PGAM5 would be improved when combining with RBDQ-HK score. Aggregates of α-synuclein in neuron are also n vital character of PD (Sadatomi et al., 2013), and in a previous study of our team, the predictive value of plasma oligomeric α-syn had been checked (Zhao et al., 2022); when plasma PGAM5 combines with oligomeric α-syn, the diagnostic utility of plasma PGAM5 would also be enhanced. However, in our study, plasma PGAM5 levels could not reflect the severity of motor symptoms (UPDRS part III score or Hoehn–Yahr stage) or non-motor manifestations of PD. Further studies are warranted to confirm the relationship between plasma levels of PGAM5 with various symptoms and the severity of PD.

There are some limitations in our study. First, we only tested PGAM5 levels in Plasma, the concentrations of PGAM5 in cerebrospinal fluid and tissues were not obtained. Second, the sample size of our study was relatively small, especially in the HC group, the accuracy of results on subgroups may be affected. Third, most disease duration of our patients was *≤*5 years (69%), and the proportion of patients in medium and advanced stages was relatively low, and this may limit further analysis on the relationship between plasma PGAM5 levels and the severity of motor or non-motor manifestations of PD. Last, this is a cross-sectional study, further longitudinal studies are warranted to confirm our findings.

## Conclusion

Plasma PAMG5 levels were significantly higher in the PD group compared with HC, with relatively high sensitivity and specificity, which suggests that plasma PGAM5 may be a diagnostic biomarker for PD, especially among elderly patients (age >60 years) and patients without HTN. Moreover, the diagnostic utility of PGAM5 would be increased when combined with plasma oligomeric α-syn or the RBDQ-HK score. Regarding motor and non-motor manifestations of PD, Plasma PAMG5 levels were found associated with the RBDQ-HK score, and further validation is warranted for its potential role as a prognostic marker for the other symptoms of PD.

## Data availability statement

The original contributions presented in the study are included in the article/supplementary material, further inquiries can be directed to the corresponding author.

## Ethics statement

The studies involving human participants were reviewed and approved by Wenzhou Medical University First Affiliated Hospital on human experimentation. The patients/participants provided their written informed consent to participate in this study.

## Author contributions

LF and HH: collected the patients and analyzed the data. XX, KX, SQ, QY, FF, and SZ: made substantial contributions to the conception and replenished the required data. CX and YL: conceived of the study and drafted the manuscript. XH: helped collect the blood samples. LF: helped revise the manuscript. All authors have read and approved the final manuscript.

## Funding

This study was supported by the Projects of the National Science Foundation of China (no. 81600977), Projects of Wenzhou City Committee of Science and Technology (Y20180137), and Projects of Natural Science Foundation of Zhejiang Province (Y19H090059).

## Conflict of interest

The authors declare that the research was conducted in the absence of any commercial or financial relationships that could be construed as a potential conflict of interest.

## Publisher’s note

All claims expressed in this article are solely those of the authors and do not necessarily represent those of their affiliated organizations, or those of the publisher, the editors and the reviewers. Any product that may be evaluated in this article, or claim that may be made by its manufacturer, is not guaranteed or endorsed by the publisher.
